# The burden of headache disorders in North India: methodology, and validation of a Hindi version of the HARDSHIP questionnaire, for a community-based survey in Delhi and national capital territory region

**DOI:** 10.1186/s10194-024-01746-x

**Published:** 2024-03-19

**Authors:** Ashish Duggal, Debashish Chowdhury, Anand Krishnan, Ritvik Amarchand, Timothy J. Steiner

**Affiliations:** 1 GB Pant Institute of Postgraduate Medical Education and Research, New Delhi, India; 2 Institute of Medical Sciences, New Delhi, India; 3 Department of Neuromedicine and Movement Science, Norwegian University of Science and Technology (NTNU), Trondheim, Norway; 4 Department of Neurology, University of Copenhagen, Copenhagen, Denmark; 5 Division of Brain Sciences, Imperial College London, London, UK

**Keywords:** Epidemiology, Global Campaign against Headache, Headache, Hindi, HARDSHIP questionnaire, India, Methodology, Population-based study, Validation

## Abstract

**Background:**

Knowledge of the prevalence and attributable burden of headache disorders in India is sparse, with only two recent population-based studies from South and East India. These produced conflicting results. A study in North India is needed. We report the methodology of such a study using, and validating, a Hindi translation of the Headache-Attributed Restriction, Disability, Social Handicap, and Impaired Participation (HARDSHIP) questionnaire developed by *Lifting The Burden* (LTB). Almost half of the Indian population speak Hindi or one of its dialects.

**Methods:**

The study adopted LTB’s standardized protocol for population-based studies in a cross-sectional survey using multistage random sampling conducted in urban Delhi and a surrounding rural area. Trained interviewers visited households unannounced, randomly selected one adult member from each and applied the Hindi version of HARDSHIP in face-to-face interviews. The most bothersome headache reported by participants was classified algorithmically into headache on ≥ 15 days/month (H15 +), migraine (including definite and probable) or tension-type headache (including definite and probable). These diagnoses were mutually exclusive. All participants diagnosed with H15 + and a 10% subsample of all others were additionally assessed by headache specialists and classified as above. We estimated the sensitivity and specificity of HARDSHIP diagnoses by comparison with the specialists’ diagnoses.

**Results:**

From 3,040 eligible households, 2,066 participants were interviewed. The participating proportions were 98.3% in rural areas but 52.9% in urban Delhi. In the validation subsample of 291 participants (149 rural, 142 urban), 61 did not report any headache (seven of those assessed by HARDSHIP, eight by headache specialists and 46 by both) [kappa = 0.83; 95% CI: 0.74-0.91]. In the remaining 230 participants who reported headache in the preceding year, sensitivity, specificity and kappa with (95% CI) were 0.73 (0.65-0.79), 0.80 (0.67-0.90) and 0.43 (0.34-0.58) for migraine; 0.71 (0.56-0.83), 0.80 (0.730.85) and 0.43 (0.37-0.62) for TTH and 0.75 (0.47-0.94), 0.93 (0.89-0.96) and 0.46 (0.34-0.58) for H15 + respectively.

**Conclusion:**

This study validates the Hindi version of HARDSHIP, finding its performance similar to those of other versions. It can be used to conduct population surveys in other Hindi-speaking regions of India.

## Introduction

Although it has been established for two decades that primary headache disorders – particularly migraine – are ubiquitous, prevalent and disabling [1–4], knowledge of the global scope and scale of headache-attributed burden is still incomplete. The series of population-based studies supported by *Lifting The Burden* (LTB) as a major component of the Global Campaign against Headache [5–7], conducted in official relations with the World Health Organisation (WHO) [8], has filled many knowledge gaps – in Western Pacific [9, 10], South East Asia [11, 12], Eastern Mediterranean [13, 14], European [15–17] and African Regions [18, 19]. All of these have used standardized methodology [20] and questionnaire [21], the latter validated in several translations [22–26]. The data from these studies have contributed to the Global Burden of Disease (GBD) study [27], so that global estimates of headache-attributed burden in successive iterations of GBD have risen over 20 years [28, 29]. But still gaps remain.

LTB’s study in the southern Indian State of Karnataka [11, 30], one of the first conducted, was noteworthy. It found a 1-year prevalence of any headache of 63.9%, with a female preponderance of 4:3 [30]. Age-standardized 1-year estimates for migraine and TTH were 25.2% and 35.1% [30], at the time considered to be substantially higher than the global means [28]. A later study from eastern India, which did not follow LTB methodology, reported a lower 1-year prevalence of migraine of 14.1% [31]. Other LTB studies [9–19] place the Karnataka estimates within the global ranges but still above the best estimates of the global means [4, 29, 32].

These conflicting estimates indicate that knowledge of the headache-attributed burden in India remains incomplete. Indeed, given the cultural, ethnic and religious diversities of India, and its sheer size, a single-state study in the south could not provide reliable estimates for the whole country [30]. This study in the north of the country, using the same protocol and questionnaire [20, 21], is therefore complementary to LTB’s study in the south [11, 30].

Estimation of the burdens attributable to specific headache types in the community by trained but non-specialist interviewers requires algorithmic diagnosis, ideally validated in each setting and language. LTB’s study in Karnataka validated and used a Kannada version of the Headache-Attributed Restriction, Disability, Social Handicap, and Impaired Participation (HARDSHIP) questionnaire [21], estimating and reporting a high sensitivity for any headache (88%), good sensitivity for migraine (63%) and tension-type headache (TTH) (57%) and excellent specificity (> 80%) for both migraine and TTH [22]. It is estimated that well over 500 million (~ 43%) of India’s population speak either Hindi or a Hindi dialect [33]. Therefore, validation of a Hindi version of HARDSHIP, while necessary for this study, will also be of help in future studies in other parts of Hindi-speaking India.

Accordingly, we report the second study in India, from the National Capital Region of Delhi (NCR). The primary aim of this study was to estimate, in the adult general population of NCR, the prevalence and attributable burden of each of the headache types of public-health importance (migraine, TTH and the group of disorders characterized by headache on ≥ 15 days/month [H15 +] including medication-overuse headache [MOH]). The enquiry also included health-care and health-service utilization for headache to inform public-health policy in NCR and India more widely. This manuscript focuses on the methodology and its adaptations.

## Methodology

### Ethics

The Institutional Ethics Committee of Maulana Azad Medical College and Associated Hospitals, New Delhi, approved the study protocol.

An information sheet describing the purpose and nature of the study, in Hindi, was presented to the prospective participants and (when appropriate) to community leaders or read out to illiterate recipients. Queries were answered. Consent was recorded from all willing participants in writing or by thumb impression.

Personal identification details were known only to the interviewers and investigators; completed questionnaires were kept in locked storage in the department of neurology at GB Pant Institute of Postgraduate Medical Education and Research (GIPMER), New Delhi.

### Study design and procedures

This was a cross-sectional population-based survey. In face-to-face interviews, the HARDSHIP questionnaire was applied by trained interviewers making unannounced visits (cold calling) to randomly selected households. Pre-pilot and pilot studies preceded the main study.

### Selection and training of interviewers

We created a field team of five members. Four were interviewers, fluent in Hindi and English and with at least high-school education as well as experience in conducting community-based surveys. The fifth, a clinical research associate (CRA) with a master’s degree in sociology, was tasked with supervision of the work of the interviewers and data entry and quality control. All attended a 1-week training programme, in Hindi, in the basic clinical features of headache and a step-by-step demonstration of completion of the questionnaire, ensuring they understood all questions and their purposes (particularly those contributing to diagnosis). This was followed by hands-on practice sessions in which the interviewers applied the questionnaire to patients and their attendants in the headache clinic at GIPMER. The filled questionnaires from these sessions were evaluated for completeness and accuracy by the CRA and investigators, with remedial training when indicated.

### Study instrument

We used the HARDSHIP questionnaire available in English [21] and in a Kannada version modified by Rao et al. [22]. Following LTB’s translation protocol for hybrid documents [34], the English version was translated into Hindi separately by a linguist and a headache specialist. The two translations were reconciled into an agreed version, before back-translation to English, with a final reconciliation to develop a Hindi version acceptable to all investigators, all fluent in Hindi.

HARDSHIP includes sociodemographic questions and neutral screening questions for headache in the lifetime and preceding year. These were answered by all participants. Those reporting headache in the last year were asked diagnostic questions based on ICHD-3 [35] (current at the time), followed by enquiries into attributable burden (lost productive time, applying the Headache-Attributed Lost Time [HALT] index [36], the impacts of headache on education, career, income, social and personal relations and family, and willingness to pay [WTP] for effective treatment). Final questions relating to headache were on health-care utilization. All these enquiries focused on the headache type that was subjectively the most bothersome in those reporting more than one type. Enquiry was also made into headache occurring on the day preceding the interview (“headache yesterday” [HY]). Questions from WHO’s 8-item quality-of-life (QoL) scale (WHOQoL-8) [37] were asked of all participants, with or without headache. Details of all these enquiries are described elsewhere [21].

### Pre-pilot and pilot studies

Following training, the field team visited a residential colony in Delhi near GIPMER, along with the investigators, to administer the questionnaire to 40 participants over two days. The purposes of this pre-pilot study were to assess ease of administration and comprehension of the questionnaire and to discover potential practical difficulties. Necessary modifications were made to the Hindi questionnaire (and notified to the institutional ethics committee).

The pilot study was performed among 40 randomly selected participants in a village in Ballabgarh (Dayalpur) that would not be part of the main study. It used, and tested, the selection procedures for the main study (households and participants), estimating the likely non-participating proportion and identifying and solving logistic problems that might hinder data collection.

### Population of interest

The population of interest for the main study was defined geographically as the adult population, aged 18–65 years, in both urban and rural areas of NCR. The region encompassed the entire State of Delhi and districts surrounding it in the contiguous States of Haryana, Uttar Pradesh and Rajasthan, with a population of over 46 million and an urbanization level of 62.6% [38].

### Sample size and sampling methodology

Anticipating 30% prevalence (the mean of migraine and TTH in Karnataka [11]), we estimated *N* = 950 to provide 3% absolute precision and α = 0.05. To afford separate estimates for urban and rural areas, we planned to include 1,000 from each (total *N* = 2,000).

We divided the urban area into low-income (slums and resettlement colonies), middle-income (flats, apartments and housing societies) and high-income colonies (bungalows) in the proportions of the total population of NCR. Using the number of rooms as a proxy measure of socioeconomic status as per the 2011 Census, we estimated 33.5% in the low-income group (≤ 1 room), 31.7% middle-income (2 rooms) and 36.9% high-income (≥ 3 rooms) [39]. Delhi had 70 assembly constituencies; we selected one assembly constituency by convenience sampling for each income group: Ambedkar Nagar (low-income), Rithala (middle-income) and Greater Kailash (high-income). Within these constituencies, we randomly selected one colony (low-, middle- and high-income respectively) using data available on the website of the Chief Electoral Officer, Delhi [40]. For the rural sample, we selected one of the non–Delhi districts in NCR, Faridabad in Haryana, and, from there, one tehsil (township), Ballabgarh, a southern neighbourhood of Faridabad. Of the 82 villages in Ballabgarh, we selected four by convenience sampling: Sunphed, Pehladpur, Deegh and Sagarpur, known to us from our earlier work there.

The sampling unit was the household, defined as a group of people living together and sharing a kitchen. For the urban areas, we took help from the Resident Welfare Associations (RWAs), obtaining lists of households in each colony and giving each a unique identifier number. In rural areas, the study team had already conducted a house-to-house census earlier that year, and each household was again given a unique number. Uninhabited or abandoned houses, institutional households (paying-guest accommodation, hostels, guest houses and commercial establishments) were excluded from these lists. From those remaining, we selected households from the numbered lists using a random number generator.

Selected households were visited unannounced. In each, when the door was opened, a responsible person was asked to list all adult members (defined as residing there for > 6 months) in a specified order: oldest male first, followed by oldest female, and so on until the youngest male and youngest female. From this list, one individual aged 18–65 years, male or female, was randomly selected using the Kish method [41]. The selected person, if available and consenting, was interviewed immediately. When he or she was not available, an appointment was made to return at a mutually convenient time. In cases of refusal, the reasons were documented. When three agreed appointments (at least two at weekends to accommodate workers) were not kept, the person was listed as a non-participant. No replacements were made within households. Persons unable to complete the interview because of physical or mental health conditions, and immigrants, were excluded from the study and not counted within the non-participating proportion.

We adopted multiple tactics to improve acceptability and promote participation. Interviewer teams included one female and one male member, each given identity cards. We enlisted the help of local primary health centres, panchayats (village councils, or groups of influential older men acknowledged by communities as their governing bodies) and other local leaders to encourage participation. When the doors of selected dwellings were locked, or no responsible person was available in the household, at least two further attempts were made before the household was excluded and replaced by the next selected from the list. When necessary, we sought the cooperation of neighbours, community leaders (sarpanch, or other members of the panchayat), or the local RWA to contact a household. In urban areas, interviewers went early in the morning or late in the evening to increase the likelihood of people being home. A final mop-up round, usually on a weekend, was conducted in a village or community where households or members had been inaccessible during earlier rounds.

### Data collection

The main study was conducted between December 2018 and June 2019. Once engaged with participants, interviewers measured blood pressure using a digital device, with the participant sitting on a chair, weight with shoes removed, using a simple portable scale, and height using a stadiometer. After these procedures, the interviewers administered the adapted and translated HARDSHIP questionnaire.

### Quality assurance

The CRA checked questionnaires for completeness, accuracy and illegible markings at the end of each day. Those requiring correction were sent back on a repeat visit to the household the next day. Regular review meetings by the investigators assessed progress and evaluated questionnaires. In field visits, investigators supervised some interviews and made random re-interviews to detect discrepancies.

### Diagnoses

The diagnostic algorithm accompanying the HARDSHIP questionnaire [21] was applied by the CRA to the most bothersome headache reported by each participant. For this, the CRA underwent additional training among outpatients of the headache clinic at GIPMER. The algorithm first identified cases of headache on ≥ 15 days/month (H15 +). Among these, participants reporting acute medication use on ≥ 15 days/month were diagnosed as probable MOH, and all others as “other H15 + ” without attempting further diagnosis since this would be unreliable at a single time-limited and unanticipated encounter [21]. All remaining participants were then classified hierarchically according to ICHD-3 [35]: definite migraine was diagnosed before definite TTH, then probable migraine and probable TTH. Any still undiagnosed were left as unclassified. Actual diagnoses will be reported in future manuscripts.

### Validation of the algorithm

Two investigators (DC and AD, both headache specialists) and two trainees in headache medicine visited the survey sites (the four villages in Ballabgarh and all urban sites) within 3 weeks of the initial interviews, evaluating, while blinded to the algorithmic diagnoses, all participants diagnosed with H15 + and a 10% subsample of all others. They first enquired about headache in the preceding year, then classified reported headaches by applying their expert knowledge and ICHD-3 criteria [35]. To improve participation in these second interviews, we organized health camps and invited participants personally and through posters and social media accounts of RWAs in urban areas. Despite these efforts, only 50% of the identified subsample of participants attended the camps. The rest were interviewed by investigators through house visits.

### Data management and statistical analysis

Data were entered by the CRA and field interviewers into Excel. Double entry of 50% of the data revealed few discrepancies (3.5%), which were resolved. A cross-check against the original questionnaires of a random 10% of the data by one of the investigators (AD) found only 0.9% errors. The data were then locked.

We compared the age and gender distributions of our sample with those of the population to assess the representativeness of the sample. For this purpose, the age-gender structure of Delhi was derived from the SRS 18 report [42]. For rural Ballabgarh, we used the data available from the census carried out in the study villages in 2018 by the study team.

Analyses were undertaken using SPSS version 25. Procedures for the main study will be reported in future papers presenting the results. In the validation analysis, we first assessed agreement between the field team and the headache specialists on the reported occurrence of headache in the preceding year. Then we compared algorithmic diagnoses derived from the field team’s enquiries with those made by the headache specialists. Using the latter as reference, we estimated sensitivity, specificity, and chance-corrected agreement (kappa) of the algorithm for any headache and each headache type. In these analyses, migraine and probable migraine were grouped as all-migraine and TTH and probable TTH as all-TTH.

## Results

### Pre-pilot and pilot studies

These studies each recruited 40 participants with no refusals. Neither study identified significant problems or predicted difficulties requiring a change of protocol for the main study. However, they did throw up uncertainties and comprehension difficulties in the proposed Hindi questionnaire. This led to the changes listed in Table 1.

**Table 1:** Changes in the Hindi version of the HARDSHIP questionnaire made prior to the main study

Change	Rationale
The response option “pricking/other” to the question on headache character was removed	This was an option in the Karnataka study [22], but not well understood by our participants, and not helpful to diagnosis
The response option “throbbing” to the question on headache character needed supplementary explanation in Hindi	This term was not well understood by our participants, but important to diagnosis
The question on headache intensity, which required evaluation against a 3-point verbal rating scale (VRS) (1: mild; 2: moderate; 3: severe), was supplemented with a 10-cm visual analogue scale (VAS)	It was easier for illiterate rural participants to describe the severity of headache on the VAS, and it validated the 3-point VRS
Modifications were made in the standardized health-care utilization section	Questions about professional consultation and investigations required adaptation to local practices
The willingness-to-pay question series was rephrased in Hindi, with the explanation that this was a hypothetical enquiry. Additionally, the interviewer first asked an open-ended question (“How much would you pay …?”). Only when a participant could not answer this was the bidding-game method employed, commencing with the modest sum of INR 2,500	Most participants found the open-ended question easier to respond to, and asking it first was time saving

### Main study

A total of 7,878 houses were enumerated in rural (1,434) and urban (6,444) areas of NCR; of these, 4,428 (rural 427; urban 4,001) were either uninhabited or commercial establishments or offices. Of 3,450 dwellings approached (rural 1,007; urban 2,443), 410 urban households were ineligible, mostly because they consisted of bachelors staying together in a house. From 3,040 eligible households visited (rural 1,007; urban 2,033), 2,066 participants were interviewed (rural 990; urban 1,076). The participating proportions were 98.3% in rural areas and 52.9% in urban areas (overall, 67.9%) (Table 2). Refusals were much higher in the high-income urban areas (67.2%) than in middle- (13.8%) or low-income (12.5%) areas. Most were because selected respondents claimed not to have time (716, 73.5%); others did not want to answer the questions (258, 26.5%). The average time taken for the interview was 45 ± SD = 28 min (range 15–65 min).

**Table 2:** Participation according to survey area and urban income group

Dwellings				Prospective participants		
Habitation (survey area) and urban income group^a^	Enumerated	Locked or ineligible	Visited and eligible (*N*)	Declined	Interviewed (*n*)	Participating proportion (*n*/*N*) (%)
Rural (Ballabgarh)	1,434	427	1,007	17	990	98.3
Urban (Delhi)	6,444	4,411	2,033	957	1,076	52.9
low-income (Ambedkar Nagar)	1,935	1,528	407	51	356	87.5
middle-income (Rithala)	684	335	349	48	301	86.2
high-income (Greater Kailash)	3,825	2,548	1,277	858	419	32.8
Total	7,878	4,838	3,040	974	2,066	68.0

^a^See text for explanation

**Fig. 1:**
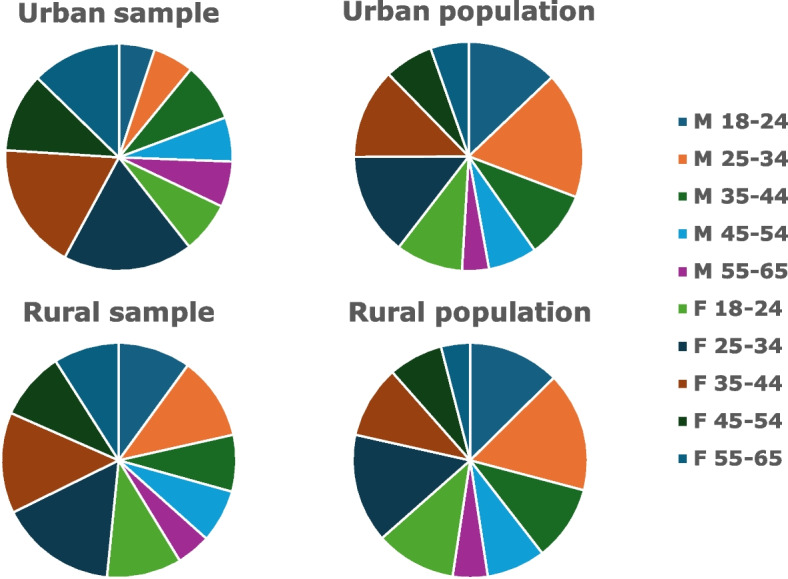
Comparison of the age-gender structures of urban and rural samples with those of the population of interest (M: male; F: female; age groups in years; also see Table 3)

Mean age of the participants was 38.8 years [median 37 years], and 64.3% were women (60% in rural and 68.3% in urban areas). Comparison of the age-gender structure of the sample with that of the population of interest showed some differences, especially in urban areas, where younger men (< 35 years) were under-represented and older women (> 45 years) over-represented in the sample (Table 3). This was also true, but to lesser extent, in the rural area. The differences are shown graphically in Fig. 1.

**Table 3:** Comparison of the age-gender structure of the sample with that of the population of interest

Category		Urban^a^		Rural^b^	
Gender	Age group (years)	Sample	Population	Sample	Population
Male	18–24	5.1	12.9	10.0	12.6
	25–34	5.8	17.9	11.5	16.5
	35–44	8.4	9.5	7.8	10.4
	45–54	6.3	6.9	7.3	8.1
	55–65	6.5	3.8	4.7	4.8
Female	18–24	7.3	9.5	10.3	11.1
	25–34	18.5	14.5	16.1	15.0
	35–44	18.1	12.8	13.9	10.0
	45–54	11.3	6.9	9.4	7.5
	55–65	12.7	5.4	9.0	4.0
		100.0	100	100.0	100.0

^a^Population structure of Delhi based on SRS 2018 age and gender estimates [42]. ^b^Population structure of rural Ballabgarh based on previous census of the study villages conducted by the study team

### Validation study

**Fig. 2:**
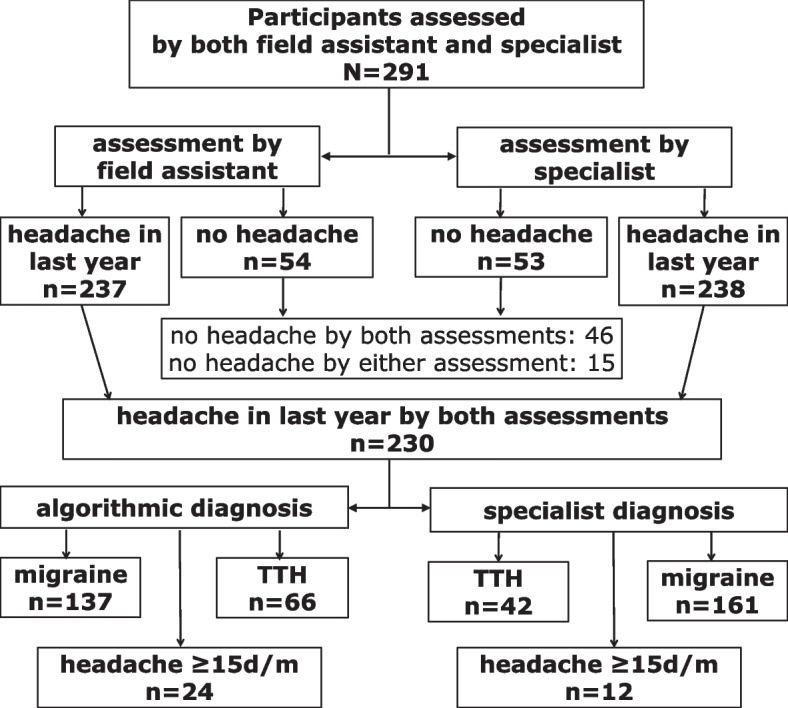
Flow diagram of participant selection for validation study (TTH: tension-type headache; d/m: days/month)

In the validation subsample of 291 participants (149 rural, 142 urban), 46 were assessed not to have any headache by both the field team and the specialists. There were 15 discordant assessments, with seven assessed to have headache by HARDSHIP only and eight by headache specialists only (kappa = 0.83; 95% CI: 0.74–0.91) (Fig. 2). For the 230 participants diagnosed with headache by both HARDSHIP and specialists, levels of agreement over headache type are shown in Table 4. The algorithm under-diagnosed migraine in favour of TTH, but nonetheless had good sensitivity and specificity for both (migraine: 0.75 and 0.81 respectively; TTH: 0.76 and 0.83) (Table 4). HARDSHIP over-diagnosed cases of H15 + according to the specialists, but still had good sensitivity and specificity (0.75 and 0.93).

**Table 4:** Comparison of algorithmic and specialist diagnoses in the validation sub-sample (*N* = 230)

Algorithmic diagnoses	Specialists’ diagnoses				Validation parameters		
	Migraine^a^	Tension-type headache^a^	Headache on ≥ 15 d/m	Totals	Sensitivity (95% CI)	Specificity (95% CI)	Kappa (95% CI)
Migraine^a^	129	8	3	143	0.75 (0.68–0.81)	0.81 (0.69–0.89)	0.46 (0.34–0.58)
Tension-type headache^a^	32	34	0	71	0.76 (0.61–0.86)	0.83 (0.77–0.88)	0.49 (0.37–0.62)
Headache on ≥ 15 d/m	12	3	9	24	0.75 (0.47–0.91)	0.93 (0.89–0.96)	0.46 (0.34–0.58)

^a^Includes probable diagnoses; d/m: days/month

## Discussion

This is the second population-based survey of headache disorders performed in India following LTB’s standardized protocol [20], and the first to employ a Hindi version of the HARDSHIP questionnaire. We found that the protocol could be readily and successfully adapted and implemented among the Hindi-speaking community of India. The validation study, reported here, showed that the Hindi-translated diagnostic question set in HARDSHIP, in careful accordance with LTB’s translation protocol for hybrid documents [34], had good sensitivity and specificity for migraine, TTH and H15 + . This was a strength of the study. Since Hindi is the most widely spoken and understood language all over India, this validation is important for future research in other areas where this language is spoken.

The performance of an algorithm is both an innate property of the algorithm and dependent on how the information it needs is collected. Language apart, the diagnostic question set in HARDSHIP is expressly intended for use by trained field interviewers, not necessarily having a medical background [21]. The moderate agreement between our trained interviewers with a basic understanding of headache on the one hand and headache specialists trained for clinical diagnostic consultation, equipped with high levels of expertise and experience and fully familiar with ICHD-3 [35] on the other, was therefore encouraging. Validation studies elsewhere with the same protocol and questionnaire have had similar experiences (Table 5), although the study in Croatia was undoubtedly influenced by its selection of University of Applied Health Sciences students as participants [26]. The sensitivity and specificity of our Hindi version of HARDSHIP for the diagnosis of migraine were comparable to those of the Russian version [23]. Sensitivity for migraine was better than that of the Kannada version used in Karnataka [22], similar to that of the Urdu version used in Pakistan [25], but less good than those in the Mandarin [24] and Croatian versions [26] (Table 5). Specificity for migraine was rather lower than in all other versions. Sensitivity for TTH was better than achieved by all versions except Croatian [26], while specificity for TTH was on a par with the Kannada version [22] but less good than achieved by the four others. Comparisons between studies of kappa values need to consider the differences in prevalence of the disorders, since, unlike sensitivity and specificity, kappa is influenced by prevalence.

**Table 5:** Comparison of performance of HARDSHIP diagnostic algorithm for migraine and tension-type headache in different studies

Study (language)	Migraine				Tension-type headache			
	Prevalence (%)	Sensitivity	Specificity	Kappa	Prevalence (%)	Sensitivity	Specificity	Kappa
Karnataka, India [22] (Kannada)	22%	0.63	0.85	0.46	33.3%	0.57	0.81	0.39
China [24] (Mandarin)	7%	0.83	0.99	0.82	14%	0.51	0.99	0.59
Russia [23] (Russian)	50%	0.77	0.82	0.58	46%	0.64	0.91	0.58
Pakistan [25] (Urdu)	21%	0.74	0.87	0.56	23.3%	0.60	0.92	0.54
Croatia [26] (Croatian)	not estimated	0.95	0.89	0.85	not estimated	0.78	0.94	0.74
Present study (Hindi)	75%	0.73	0.80	0.43	19%	0.71	0.80	0.43

There are multiple contributors to diagnostic uncertainty. Respondents to a survey are likely to take a more casual approach to diagnostic enquiry than those in consultation with a headache specialist, the latter having more prospect of personal benefit. One of the crucial factors in this survey, as in the use of HARDSHIP generally [19], was the identification of and focus upon the most bothersome headache. The subjective evaluation this requires may be influenced by factors that are inconstant over relatively short periods of time, such as the most recent headache. The generally stoical nature of Indians, especially women and those from rural areas, disincline them to complain of severe or disabling headache; indeed, “inability” to function itself has a subjective and context-dependent element, and may be overridden by absolute need to function when no help is available.

The ICHD criteria, on which algorithmic diagnosis must be based, themselves have imperfect (and currently unknowable) sensitivity and specificity for the headache types, being largely opinion-based in the absence of clear biomarkers [35]. While generally they have emphasis on specificity over sensitivity [35, 43], they have particular built-in insensitivity for TTH because of its lack of specific characteristics [35]. The distinction between migraine and TTH may hinge on the subjective judgment of headache intensity on an insensitive scale (1–3), on the difference between throbbing and pressing, or on the occurrence or not of photophobia and phonophobia, which are conceptually problematic to lay people and notoriously difficult to express in lay-worded questions [21]. The distinction between both migraine and TTH on the one hand and H15 + on the other rests, in questionnaire diagnoses, on a simple frequency count based on sometimes unreliable recall. Questionnaires cannot engage in the judgments required to separate chronic forms of migraine and TTH [21]. Headache specialists can, although often only with deep probing.

Our sampling method, interviewing in both rural and urban areas and across the socioeconomic range, aimed to ensure geographic and socioeconomic representativeness of the region. Despite this, our final sample was significantly different from the population of interest in both areas, although more so in urban. Our considerable efforts to encourage participation ensured a high participating proportion (98.4%) in rural areas but were far less successful in urban, particularly among the high-income colonies. Here, 957 (47.1%) prospective participants refused to be part of the survey either because of claimed lack of time (707) or because they did not wish to engage in the survey (250). People in higher-income areas are less dependent on the public-health system and may reasonably be apathetic towards surveys that may not be of benefit to them. Some had issues regarding privacy of data, and some were reluctant to share their medical details despite our assurances of rigorous data protection. Overall, this low participation among a particular sector of the population, despite our best efforts, will have to be recognized as one of the weaknesses of the study, albeit probably unavoidable, when the full results are presented.

## Conclusions

This study successfully validated the Hindi version of LTB’s HARDSHIP questionnaire [21]. Careful multistage random sampling to ensure representativeness proved imperfect, largely because of selective non-participation, a potential and to an extent unavoidable hazard in all population-based studies. Otherwise, we found that LTB’s standardized methodology for estimating the prevalence of headache disorders worked well in this region of northern India. The results of the full survey, to be presented in later papers, can be compared with those from the similar study in Karnataka in southern India [11, 30], while the Hindi-translated instrument will be available for studies in other Hindi-speaking areas of India.

## Data Availability

Electronic data are held securely at GB Pant Institute of Postgraduate Medical Education and Research. When analyses are completed, anonymised data will be available on request for academic purposes.

## Abbreviations

CRA: Clinical research associate
GBD: Global Burden of Disease
GIPMER: GB Pant Institute of Postgraduate Medical Education and Research
HARDSHIP: Headache-Attributed Restriction, Disability, Social Handicap and Impaired Participation (questionnaire)
H15 +: Headache on ≥ 15 days/month
HALT: Headache-Attributed Lost Time (index)
ICHD: International Classification of Headache Disorders
LTB: *Lifting The Burden*
MOH: medication-overuse headache
NCR: National Capital Region of Delhi
QoL: Quality of life
RWA: Resident Welfare Association
TTH: Tension-type headache
VAS: Visual analogue scale
VRS: Verbal rating scale
WHO: World Health Organization
